# Numerical Study of the Effect of Thixotropy on Extrudate Swell

**DOI:** 10.3390/polym13244383

**Published:** 2021-12-14

**Authors:** Michelle Spanjaards, Gerrit Peters, Martien Hulsen, Patrick Anderson

**Affiliations:** 1 Department of Mechanical Engineering, Eindhoven University of Technology, P.O. Box 513, 5600 MB Eindhoven, The Netherlands; m.m.a.spanjaards@tue.nl (M.S.); g.w.m.peters@tue.nl (G.P.); m.a.hulsen@tue.nl (M.H.); 2 VMI Holland B.V., Gelriaweg 16, P.O. Box 161, 8161 RK Epe, The Netherlands

**Keywords:** viscoelasticity, thixotropy, extrudate swell, FEM

## Abstract

The extrusion of highly filled elastomers is widely used in the automotive industry. In this paper, we numerically study the effect of thixotropy on 2D planar extrudate swell for constant and fluctuating flow rates, as well as the effect of thixotropy on the swell behavior of a 3D rectangular extrudate for a constant flowrate. To this end, we used the Finite Element Method. The state of the network structure in the material is described using a kinetic equation for a structure parameter. Rate and stress-controlled models for this kinetic equation are compared. The effect of thixotropy on extrudate swell is studied by varying the damage and recovery parameters in these models. It was found that thixotropy in general decreases extrudate swell. The stress-controlled approach always predicts a larger swell ratio compared to the rate-controlled approach for the Weissenberg numbers studied in this work. When the damage parameter in the models is increased, a less viscous fluid layer appears near the die wall, which decreases the swell ratio to a value lower than the Newtonian swell ratio. Upon further increasing the damage parameter, the high viscosity core layer becomes very small, leading to an increase in the swell ratio compared to smaller damage parameters, approaching the Newtonian value. The existence of a low-viscosity outer layer and a high-viscosity core in the die have a pronounced effect on the swell ratio for thixotropic fluids.

## 1. Introduction

In the automotive industry, rectangular rubber strips are extruded that are used to make the carcass of car tires. The dimensions and quality of the rubber extrusion products highly depend on the rheological properties of the rubber compound [1]. These compounds are complex materials in the sense that they contain many additives such as plasticizers, curing agents and about 30% by weight of reinforcing fillers to enhance the mechanical properties of the final product [2].

The addition of fillers increases the viscosity of the compound due to the existence of filler–filler and polymer–filler interactions and is essential to the successful use of rubber in the extrusion process [3,4]. When using carbon black fillers, the primary filler particles form aggregates, and the size and shape of these aggregates are deformation-independent. These aggregates, however, can cluster together to form agglomerates, which can form a filler–filler network that is held together by weak van der Waals-type forces. Because of the fragility of the bonds between the agglomerates, they can break under stress, but when the stress is removed, these bonds will reform again [5]. This leads to a reversible decrease in the viscosity, or so-called thixotropic behavior. For rubber compounds, this is also known as the Payne effect [6].

The Payne effect has also been attributed to several other mechanisms, such as the agglomeration/deagglomeration of filler aggregates, breakup/reformation of the filler–filler and polymer–filler network [7,8], chain desorption from the fillers [9], yielding of the glassy layer between the fillers [10] and disentanglement of the absorbed chains [11]. The paper by Rueda et al. reviews the current knowledge about the rheology and applications of highly filled polymers [12].

Dangtungee et al. [13,14] experimentally studied the extrudate swell of polypropylene filled with different weight percentages of ${\mathrm{CaCO}}_{3}$ and ${\mathrm{TiO}}_{2}$ nano-particles. They found that swell was reduced by increasing particle concentration. This was explained by the limited mobility of the polymers due to the fillers, hindering the elastic recovery at the die exit. For an LDPE filled with different weight percentages of salt of different sizes, it was also found that the rigid particles lead to a decreased mobility of the polymer chains. No effect of the particle size was found [15].

In highly-filled rubber compounds, a decrease in extrudate swell can be observed with increasing filler content [16,17,18]. This reduction also occurs if the reinforcing character of the carbon-black is increased or the particle size is decreased and was attributed to a higher volume fraction of “occluded rubber”. Here, the fillers reduce the mobility of the polymer chains, prohibiting the elastic recoil of the polymer chains. For highly reinforcing carbon-blacks, extrudate swell is restrained by a more complex mechanism due to the complex compound morphology.

Since thixotropy has a pronounced effect on the viscosity of the material, bands of different viscosities can coexist in the die during the extrusion process. Due to the high shear rate at the die wall, a low viscous layer will be present there. The inelastic theory of extrudate swell presented by Tanner [19] showed that a less viscous outer layer results in a decreasing swell ratio that can even go below one. This was also found by Mitsoulis [20] in extrudate swell studies for double-layer flows.

The aim of this work is to qualitatively study the effect of thixotropy on the extrudate swell behavior of viscoelastic materials using the Finite Element Method. This is a relevant problem since the extrusion of filled rubber compounds is widely used in the automotive industry and the thixotropic behavior due to the incorporation of these fillers has a pronounced effect on the shape of the final extrusion product. Therefore, we assume that the undamaged material resembles a highly filled polymer filled with agglomerates of an elastic filler–filler/filler–polymer network. The weak physical bonds linking adjacent filler agglomerates can break up when the material is deformed, leading to a material with the properties of a highly filled polymer with less structure compared to the undamaged material. The disappearance of this structure effectively reduces the elasticity in the material. This thixotropic effect due to the added fillers is modeled using a structure parameter that indicates the degree of local structure in the material. The evolution of this structure parameter is modeled using a rate and stress-controlled kinetic equation. The difference between both approaches is discussed. In many industrial processes, the flow rate is not constant but fluctuates in time. Therefore, the effect of structure damage and recovery on extrudate swell is studied for a constant and fluctuating flow rate.

The paper is structured as follows: first, the problem definition and a description of the modeling is given in Section 2. This is followed by a detailed explanation of the numerical method used in this work in Section 3. A convergence study and the results for the constant flow rate and fluctuating flow rate are presented in Section 4. Here, the difference in the results for a stress and a rate-controlled kinetic equation for the structure parameter are discussed, as well as the influence of different damage and recovery parameters on the swell ratio of the extrudate.

## 2. Problem Description

Two problems are treated in this paper: a 2D planar swell problem and the swell of a 3D rectangular extrudate, both for a thixotropic fluid.

### 2.1. 2D Planar Problem

**Figure 1:**
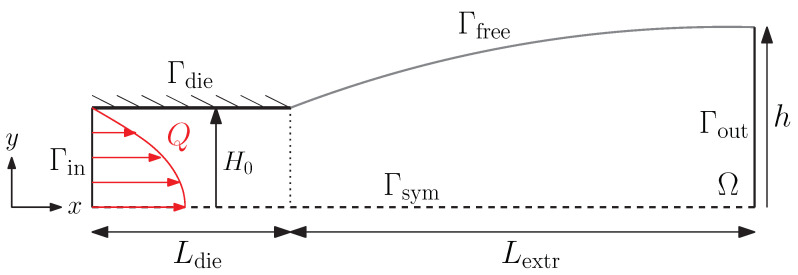
Schematic representation of the 2D planar extrudate swell problem. The free surface of the extrudate is indicated in gray.

For the 2D planar problem, a schematic representation of the fluid domain Ω is shown in Figure 1. In the first part of the domain, the fluid is contained in a planar die with half-height $H_{0}$ and length $L_{\mathrm{die}}=2H_{0}$. At the inlet of the die, a flow rate *Q* is applied with a fully developed flow profile. The extrudate has length $L_{\mathrm{extr}}=5H_{0}$.

### 2.2. Three-Dimensional Problem

**Figure 2:**
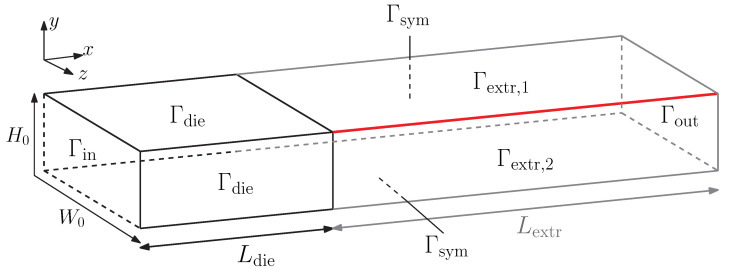
Schematic representation of the 3D problem of an extrudate emerging from a rectangular die. The corner line used in the corner-line method is depicted in red.

For the 3D rectangular extrudate, a schematic representation of the fluid domain Ω is shown in Figure 2.

The first part of the domain is the fluid contained in a rectangular die of height $H_{0}$ and width $W_{0}$. A constant flow rate *Q* is applied at the inlet $\Gamma_{\mathrm{in}}$ of the die. After length $L_{\mathrm{die}}=2H_{0}$, the fluid exits the die. The extrudate is modeled for a length $L_{\mathrm{extr}}=5H_{0}$ after the die exit. The corner line of the extrudate, used in the corner-line method as presented by Spanjaards et al. [21], is indicated in red. Only a quarter of the domain is modeled to save computational costs. The rectangular die has an aspect ratio of 2:1 with $W_{0}=2H_{0}$.

### 2.3. Balance Equations

It is assumed that the fluid is incompressible, inertia can be neglected and that there are no external body forces acting on the fluid. This leaves the following equations for the mass and momentum balance in the fluid domain Ω:

(1) $$\begin{array}{c} -\nabla \cdot \sigma =0\;\mathrm{in}\;\Omega , \end{array}$$

(2) $$\begin{array}{c} \;\;\;\nabla \cdot u=0\;\mathrm{in}\;\Omega , \end{array}$$

where u is the fluid velocity and σ is the Cauchy stress tensor:

(3) $$\sigma =-pI+2\eta_{s}D+\tau .$$

Here, *p* is the pressure, I the unit tensor and $2\eta_{s}D$ is the Newtonian (or viscous) stress tensor with solvent viscosity $\eta_{s}$ and rate-of-deformation tensor $D=(\nabla u+\nabla u^{T})/2$. The viscoelastic stress tensor is represented by τ.

### 2.4. Constitutive Equations

The viscoelastic stress tensor is expressed in terms of the conformation tensors $c_{k}$:

(4) $$\tau =\sum_{k=1}^{m}G_{k}\left(c_{k}-I\right),$$

where *m* is the number of modes and $G_{k}$ is the polymer modulus of mode *k*.

The evolution of the conformation tensors $c_{k}$ is given by

(5) $$\frac{Dc_{k}}{Dt}-{\left(\nabla u\right)}^{T}\cdot c_{k}-c_{k}\cdot \nabla u+f\left(c_{k}\right)=0,$$

where D()/Dt=∂()/∂t+u·∇() denotes the material derivative, and $f(c_{k})$ depends on the constitutive model used. In this paper, the Giesekus model is used [22]:

(6) $$f\left(c_{k}\right)=\frac{1}{\lambda_{k}}\left[c_{k}-I+\alpha_{k}{\left(c_{k}-I\right)}^{2}\right],$$

where $\alpha_{k}$ is the mobility parameter of mode *k* that influences shear-thinning. Other constitutive equations can be used due to the generality of our method.

### 2.5. Thixotropy Model

In thixotropic materials, it is known that the added fillers can form two types of networks: filler–filler networks and polymer–filler networks. These networks improve the strength of the material. These networks are held together by weak physical bonds. When the material is deformed, these bonds can break, but after flow cessation, these bonds can reform again. To model this thixotropic behavior, a structure parameter ξ is defined which indicates the degree of structure in the material, as discussed in Spanjaards et al. [23]. If ξ=1, the material is undamaged and the filler–filler/polymer–filler networks are intact. For $\xi =\xi_{\inf}$, no network structures are left:

$$\xi =\left\{\begin{array}{cc} 1, & \;\mathrm{Undamaged};\;\mathrm{structures}\;\mathrm{intact}\\ \xi_{\inf}, & \;\mathrm{Maximum}\;\mathrm{damage}. \end{array}\right.$$

For the minimum value for $\xi_{\inf}=0$, no structure is left. Inspired by the Leonov modeling [24], the effect of structure damage is modeled by adjusting the relaxation times of the undamaged spectrum $\lambda_{0,k}$ (ξ=1) with the current structure parameter:

(7) $$\lambda_{k}=\xi \lambda_{0,k}.$$

The polymer modulus $G_{k}$ is assumed to be independent of the structure change in the material. Notice that, using this approach, the polymer viscosity $\eta_{p,k}\left(\xi \right)$ and the relaxation time $\lambda_{k}\left(\xi \right)$ of mode *k* depend on the structure according to $\eta_{p,k}\left(\xi \right)=G_{k}\lambda_{k}\left(\xi \right)$. The ratio between the solvent viscosity and the zero-shear viscosity for the undamaged material is defined as $\beta_{0}=\eta_{s}/\eta_{0}$. Here, $\eta_{0}=\eta_{s}+\eta_{p0}$ is the zero-shear viscosity, with $\eta_{p0}=\sum_{k=1}^{m}\eta_{p0,k}$ being the total polymer viscosity of the undamaged material.

The evolution of the structure parameter can be described with a rate or stress-controlled kinetic equation. The rate-controlled equation is as follows [24]:

(8) $$\frac{D\xi }{Dt}=\frac{1-\xi }{\lambda_{\theta }}-E\gamma^{*}\left(\xi -\xi_{\inf}\right),$$

where $\lambda_{\theta }$ is a characteristic time scale for the recovery of the material structure, $E=\sqrt{2\mathrm{tr}D^{2}}$ is a measure of the deformation rate based on the rate of deformation tensor D, corresponding to the shear rate in shear flows. Furthermore, $\gamma^{*}$ is a dimensionless fitting parameter that indicates how much of the applied deformation leads to the damage of the structure. We modified Equation (8) to obtain the following stress-controlled equation:

(9) $$\frac{D\xi }{Dt}=\frac{1-\xi }{\lambda_{\theta }}-\frac{\tau_{c}\left(\tau \right)}{\eta_{p0}}\tau^{*}\left(\xi -\xi_{\inf}\right),$$

where $\tau_{c}\left(\tau \right)$ is a characteristic stress in the material that is a function of the viscoelastic stress tensor, and $\tau^{*}$ is a dimensionless fitting parameter that describes how much of the present stress contributes to the damage of the elastic network. Here, the equivalent von Mises shear stress is used as characteristic stress $\tau_{c}\left(\tau \right)$ [25]:

(10) $$\tau_{c}\left(\tau \right)=\sqrt{\frac{1}{2}\hat{\tau }:\hat{\tau }},$$

with $\hat{\tau }=\tau -\frac{1}{3}\left(\mathrm{tr}\;\tau \right)I$, the deviatoric part of the total viscoelastic stress tensor.

### 2.6. Arbitrary Lagrangian–Eulerian Formulation

For both the 2D planar and 3D swell problem, a body-fitted approach is used to take into account the movement of the free surfaces. To this end, the domain is described with a mesh that is moving in time in such a way that it follows the movement of the free surfaces, but not necessarily the movement of the fluid. Therefore, the governing equations are rewritten in the Arbitrary Lagrangian–Eulerian (ALE) formulation [26]. The convective terms in equations that contain material derivatives have to be corrected for the mesh movement:

(11) $$\frac{D()}{Dt}=\frac{\partial ()}{\partial t}{|}_{x_{g}}+\left(u-u_{m}\right)\cdot \nabla \left(\right).$$

where $\partial \left(\right)/\partial t{|}_{x_{g}}$ denotes the time derivative at a fixed grid point $x_{g}$ and $u_{m}$ is the mesh velocity.

### 2.7. Free Surface Description

#### 2.7.1. Two-Dimensional Planar Problem

For the 2D planar problem, the evolution of the free surface is described using a 1D height function [27]:

(12) $$\frac{\partial h}{\partial t}+u_{x}\frac{\partial h}{\partial x}=u_{y},$$

where *h* is the height of the free surface in every node on $\Gamma_{\mathrm{free}}$ and the subscript *y* indicates the swell direction of the free surface.

#### 2.7.2. Three-Dimensional Problem

For the 3D problem, the corner-line method as described in [21] is used to obtain the positions of the free surfaces. Here, the corner lines of the extrudate are described as material lines. The following kinematic equation is solved to obtain the *y* and *z*-positions of these lines:

(13) $$\frac{\partial d}{\partial t}+u_{x}\frac{\partial d}{\partial x}=u_{2D},$$

where d is the position vector containing the positions *f* in *y* and *z*-directions $d=(f_{y},\;f_{z})$, and $u_{2D}$ is the velocity vector containing the velocities in *y* and *z*-directions $u_{2D}=\left(u_{y},\;u_{z}\right)$.

The free surfaces, connected by a corner line, are described using 2D height functions [27]. The domain of the height function is not constant in time but changes due to the movement of the corner lines. This change has to be taken into account, and this is done using the ALE method. This leads to the following equation to obtain the heights *h* of the free surfaces:

(14) $$\frac{\partial h}{\partial t}{|}_{x_{g}}+u_{x}\frac{\partial h}{\partial x}+\left(u_{z}-u_{m,z}\right)\frac{\partial h}{\partial z}=u_{y},$$

where $\partial \left(\right)/\partial t{|}_{x_{g}}$ denotes the time derivative in a fixed grid point of the 2D grid of the expanding domain, the subscript *z* indicates the direction of the expanding 2D (*x*,*z*) domain, and $u_{m,z}$ is the corresponding mesh velocity. The subscript *y* indicates the swell direction of the upper free surface (see Figure 2). For the free surfaces at the sides of the die, *y* and *z* in Equation (14) are interchanged due to the rotation of the surface with respect to the upper free surfaces.

### 2.8. Boundary- and Initial Conditions

Schematic representations of the 2D and 3D domains are shown in Figure 1 and in Figure 2, respectively. Fully developed inflow conditions are prescribed at the inlet boundary $\Gamma_{\mathrm{in}}$ by first solving a subproblem of a periodic channel. A flow rate *Q* is enforced to this channel as a constraint using a Lagrange multiplier. The periodic velocity, structure parameter and conformation tensor solution of this channel are prescribed as an essential boundary condition to the inlet boundary ($\Gamma_{\mathrm{in}}$) of the problem. Note, that the periodic solution is a function of time *t*. At the walls of the die ($\Gamma_{\mathrm{die}}$), a no-slip boundary condition is applied, whereas the tractions are zero at the free surfaces ($\Gamma_{\mathrm{free}}$). At the outlet ($\Gamma_{\mathrm{out}}$), a free outflow is described, which means that there is no velocity in *y* and *z*-directions and the traction in *x*-direction is zero. The boundary conditions are given by

(15) $$\begin{array}{cccc} \; & u_{\mathrm{in}}=u_{\mathrm{chan}}\; & \; & \mathrm{on}\;\Gamma_{\mathrm{in}},\\ \; & c_{k,\mathrm{in}}=c_{k,\mathrm{chan}}\; & \; & \mathrm{on}\;\Gamma_{\mathrm{in}},\\ \; & \xi_{\mathrm{in}}=\xi_{\mathrm{chan}}\; & \; & \mathrm{on}\;\Gamma_{\mathrm{in}},\\ \; & u=0\; & \; & \mathrm{on}\;\Gamma_{\mathrm{die}},\\ \; & u_{y}=0\; & \; & \mathrm{on}\;\Gamma_{\mathrm{out}},\\ \; & t_{x}=0\; & \; & \mathrm{on}\;\Gamma_{\mathrm{out}},\\ \; & t=0\; & \; & \mathrm{on}\;\Gamma_{\mathrm{free}}, \end{array}$$

where $u_{\mathrm{chan}}$, $\xi_{\mathrm{chan}}$ and $c_{k,\mathrm{chan}}$ are obtained from the separate periodic channel problem. The traction vector on the surface with an outwardly directed normal n is denoted by t=σ·n. An essential boundary condition on the height function of every free surface is applied such that the free surface stays attached to the die.

The initial conditions for the height functions are given by

(16) $$\begin{array}{cc} \; & d\left(t=0\right)=d_{0},\\ \; & h\left(t=0\right)=H_{0}, \end{array}$$

where $d_{0}$ and $H_{0}$ are equivalent to the coordinates of the corner points of the die and the height of the die, respectively. The initial condition for the conformation tensor $c_{k}$ in Equation (5) is given by

(17) $$c_{k}\left(t=0\right)=I,$$

The fluid is initially assumed to be undamaged, which leads to the following initial condition for the structure parameter ξ:

(18) ξ(t=0)=1

The initial conditions presented in Equations (17) and (18) are applied to both the periodic inlet channel and Ω.

## 3. Numerical Method

The finite element method is used to solve the governing equations. The log-conformation representation [28], SUPG [29] and DEVSS-G [30] are used for stability in solving the constitutive equation. SUPG is also used for stability in the height function equations of the free surfaces and the corner lines.

### 3.1. Weak Formulations

The weak formulation of the balance equations can be derived by multiplying the equations with test functions and integrating over the domain using partial integration and the Gauss theorem.

The weak form of the mass and momentum balance and the constitutive equation can now be formulated as follows: find u, *p*, G and $s_{k}$ such that

(19) $$\begin{array}{cc} \; & \left({\left(\nabla v\right)}^{T},\nu \left(\nabla u-G^{T}\right)\right)+\left(D_{v},2\eta_{s}D+\tau \right)-\left(\nabla \cdot v,p\right)=0, \end{array}$$

(20) $$\begin{array}{cc} \; & (q,\nabla \cdot u)=0, \end{array}$$

(21) $$\begin{array}{cc} \; & (H,-\nabla u+G^{T})=0, \end{array}$$

(22) $$\begin{array}{cc} \; & \left(\zeta +\tau_{1}\left(u-u_{m}\right)\cdot \nabla \zeta ,\frac{\partial s_{k}}{\partial t}{|}_{x_{g}}+\left(u-u_{m}\right)\cdot \nabla s_{k}-g\left(G,s_{k}\right)\right)=0, \end{array}$$

for all admissible test functions v,q,H,ζ. Furthermore, $D_{v}=\left(\nabla v+{\left(\nabla v\right)}^{T}\right)/2$, (·,·) denotes the inner product on domain Ω, and ν and $\tau_{1}$ are parameters due to DEVSS-G and SUPG stabilization, respectively. Furthermore, $s_{k}=\log c_{k}$. More information on log-conformation stabilization and the function g can be found in [28], whereas more information on the DEVSS-G method and the projected velocity gradient G can be found in [30].

The weak form of the evolution equations for the rate and stress-controlled structure parameter can be formulated as follows: find ξ such that

(23) $$\left(\chi +\tau_{2}u\cdot \nabla \chi ,\frac{\partial \xi }{\partial t}{|}_{x_{g}}+\left(u-u_{m}\right)\cdot \nabla \xi -\frac{1-\xi }{\lambda_{\theta }}+f\left(\xi -\xi_{\inf}\right)\right)=0$$

for admissible test functions χ. Here, $f=E\gamma^{*}$ for the rate-controlled model, $f=\left(\tau_{c}\tau^{*}\right)/\eta_{p0}$ for the stress-controlled model, and $\tau_{2}$ is again a parameter due to SUPG stabilization. For the 2D problem, the weak formulation for the height function is the same as formulated by Choi and Hulsen [31], whereas for the 3D problem, the weak formulations of the height functions of the corner lines and the free surfaces can be found in [21].

### 3.2. Spatial Discretization

For the 2D planar isoparametric problem, triangular $P_{2}P_{1}$ (Taylor–Hood) elements are used for the velocity and pressure. For the conformation, triangular $P_{1}$ elements are used. For the 1D height function, quadratic line elements are used, whereas for the kinetic equations of the structure parameter, triangular $P_{1}$ elements are used. A structured mesh is generated using Gmsh [32]. For the 3D isoparametric problem, tetrahedral $P_{2}P_{1}$ (Taylor–Hood) elements are used for the velocity and pressure, whereas for the conformation, tetrahedral $P_{1}$ elements are used. For the 1D height functions of the corner lines, quadratic line elements are used, whereas for the 2D height functions of the free surfaces, quadratic triangular elements are used. For the kinetic equations of the structure parameter, tetrahedral $P_{1}$ elements are used. Equations (22) and (23) are solved using SUPG for stability. The SUPG parameters are obtained as follows:

(24) $$\tau =\beta_{\mathrm{SUPG}}\frac{h_{\mathrm{elem}}}{2|u|}.$$

where $\beta_{\mathrm{SUPG}}=1$, τ is calculated in every integration point and $h_{\mathrm{elem}}$ is the element size and is defined using the method of Hughes et al. [33]. SUPG stabilization is also used for the height function of the free surface. More information on the SUPG parameter for the weak form of the height function for the 2D planar problem can be found in [31], whereas for the 3D problem, this can be found in [21].

### 3.3. Time Discretization

A predictor–corrector scheme is used to obtain the positions of the free surface. To start, a Newtonian time step is performed with an initially homogeneous undamaged structure parameter field ξ=1 to obtain the initial velocities and pressures. The numerical procedure of every time step is now as follows:

**Step** **1** Predict and update the position of the free surface, $x_{\mathrm{free}}$, in the bulk mesh. For the first time step, the prediction of the position equals the initial position: $x_{\mathrm{free},\mathrm{pred}}=x_{\mathrm{free},0}$. For subsequent time steps, a second-order prediction of the free surface position is used:
  (25) $$x_{\mathrm{free},\mathrm{pred}}=2x_{\mathrm{free}}^{n}-x_{\mathrm{free}}^{n-1}.$$
**Step** **2** Construct the ALE mesh. This is done by solving a Laplace equation to obtain the mesh displacement, as explained in [21]. The new coordinates of the nodes are calculated using this obtained mesh displacement.
**Step** **3** The mesh velocities can now be obtained by numerically differentiating the mesh displacement. In the first time step, the mesh velocities are zero, since the height function is equal to the initial height $H_{0}$. For subsequent time steps, a second-order backward differencing scheme is used, using the updated mesh nodes:
  (26) $$u_{m}^{n+1}=\frac{\frac{3}{2}x_{m}^{n+1}-2x_{m}^{n}+\frac{1}{2}x_{m}^{n-1}}{\Delta t},$$
  where Δt is the time step used.
**Step** **4** A prediction is done for the velocity and the conformation fields. In the first time step, a first-order prediction is used: $\hat{u}=u^{n}$, $\hat{c}=c^{n}$. For subsequent time steps, a second-order prediction of the velocity and conformation field is used:
  (27) $$\begin{array}{c} \hat{u}=2u^{n}-u^{n-1}, \end{array}$$
  (28) $$\begin{array}{c} \hat{c}=2c^{n}-c^{n-1}. \end{array}$$
  The velocity prediction is used to calculate $E^{n+1}$ in the rate-controlled kinetic equation for ξ, whereas the conformation prediction is used to calculate the von Mises equivalent shear stress $\tau_{c}\left(\hat{\tau }\right)$, as given by Equation (10), in the stress-controlled equation for ξ. Equation (23) can now be solved to obtain the structure parameter $\xi^{n+1}$ in every node of the mesh. For the first time step, first-order time integration is used:
  (29) $$\frac{\partial \xi }{\partial t}{|}_{x_{g}}=\frac{\xi^{n+1}-\xi^{n}}{\Delta t},$$
  whereas for subsequent time steps, second-order time integration is used:
  (30) $$\frac{\partial \xi }{\partial t}{|}_{x_{g}}=\frac{\frac{3}{2}\xi^{n+1}-2\xi^{n}+\frac{1}{2}\xi^{n-1}}{\Delta t}.$$
  The relaxation times are now updated using $\xi^{n+1}$.
**Step** **5** Using the method of D’Avino and Hulsen [34] for decoupling the momentum balance from the constitutive equation, the velocities $u^{n+1}$ and pressures $p^{n+1}$ are computed. Using this implicit stress formulation, the balance equations are solved using a prediction for the viscoelastic stress tensor to find $u_{n+1}$ and $p_{n+1}$ at every time step.
**Step** **6** After solving for the new velocities and pressures, the actual conformation tensor $c^{n+1}$ is found using a second-order, semi-implicit extrapolated backward differencing scheme with conformation prediction for Equation (22).
**Step** **7** Update the position of the free surface by solving the evolution equation of the height function (12). For the first time step, first-order time integration is used, whereas for subsequent time steps, second-order time integration is used, as explained in [31].

For the 3D problem, the time integration scheme as presented in [21] is used. The structure parameter ξ is calculated in the same way as presented in the time integration scheme in this section.

## 4. Results

First, the results for mesh and time convergence are shown, followed by results for the extrudate swell of thixotropic fluids. The relevant parameters used throughout this work are given in Table 1. From now on, we refer to $\gamma^{*}$ and $\tau^{*}$ as the “damage parameters”.

where $\lambda_{\mathrm{avg}}=\left(\sum_{k=1}^{m}G_{0,k}\lambda_{0,k}^{2}\right)/\left(\sum_{k=1}^{m}\eta_{p0,k}\right)$ is the viscosity averaged relaxation time of the undamaged material, with *m* the number of modes. The Weissenberg number of the problem is defined as follows:

(31) $$\mathrm{Wi}=\frac{U_{\mathrm{avg}}\lambda_{\mathrm{avg}}}{H_{0}}$$

This spectrum is chosen because it represents an elastic material with strong shear-thinning behavior, as is characteristic for rubber compounds [35].

### 4.1. Convergence

To verify if the rate and stress-controlled thixotropy models are correctly implemented, a convergence study is performed. A convergence study of the 2D and 3D swell code was performed by Spanjaards et al. in [21,36]. Therefore, we now focus on the implementation of the thixotropy models. In this convergence study, the thixotropy model is decoupled from the flow, which means that the relaxation times are not adjusted with the structure parameter ξ. A channel flow with length $L=100H_{0}$ of a single-mode Upper Convected Maxwell (UCM) fluid (with relaxation time $\lambda_{0}$ and $\eta_{s}=0$) is modeled. A flow rate *Q* is applied at the channel inlet, and the analytical solution of a fully developed flow is prescribed to the velocity and the viscoelastic stress tensor. The Weissenberg number of the problem equals Wi=1. For a fully developed channel flow of an UCM fluid, the velocity, shear rate and viscoelastic stresses can be found to be

(32) $$u_{x}\left(y\right)=\frac{3Q}{2H_{0}}\left(1-\frac{y^{2}}{H_{0}^{2}}\right)$$

(33) $$\dot{\gamma }=\frac{3Q}{H_{0}^{3}}y,$$

(34) $$\sigma_{xx}=2\eta_{0}{\dot{\gamma }}^{2},\;\sigma_{yy}=0,\;\sigma_{xy}=\eta_{0}\dot{\gamma },$$

where $H_{0}$ is the half height of the channel, $\eta_{0}$ is the zero-shear viscosity of the fluid, *Q* is the flow rate applied at the inlet of the channel, and *y* is the *y*-coordinate of the height of the channel. Analytical solutions of Equations (8) and (9) can be defined as follows:

(35) $$\xi_{\mathrm{an}}=Ae^{-(\frac{1}{\lambda_{\theta }}+f)t}+\xi_{\mathrm{eq}},$$

with

(36) $$A=1-\frac{1+f\lambda_{\theta }\xi_{\inf}}{1+f\lambda_{\theta }}.$$

where *f* and $\xi_{\mathrm{eq}}$ can be expressed as follows for the rate and stress-controlled equation, respectively:

(37) $$f=\dot{\gamma }\gamma^{*}\;\;\left(\mathrm{rate}\right),\;f=\frac{\tau_{c}\left(\tau \right)}{\eta_{0}}\tau^{*}\;\;\left(\mathrm{stress}\right),$$

(38) $$\xi_{\mathrm{eq}}=\frac{1+f\lambda_{\theta }\xi_{\inf}}{1+f\lambda_{\theta }}.$$

**Figure 3:**
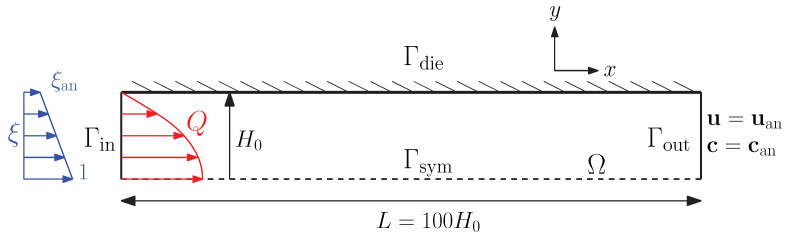
Schematic representation of the 2D channel problem used in the convergence study.

To avoid a singular point at the die wall, a linear profile for the structure parameter ξ is applied as an essential boundary condition at the inlet. At the die wall, the analytical solution for ξ is prescribed while linearly decreasing to zero with the *y*-coordinate at the symmetry axis of the channel. A schematic representation of the 2D channel problem is shown in Figure 3.

#### 4.1.1. Mesh Convergence

The solution of $\xi_{\mathrm{an}}$ for meshes with different element sizes is compared to the computed ξ at the outlet of the channel $\Gamma_{\mathrm{out}}$ for the rate and stress-controlled implementation. More information on the meshes used can be found in Table 2. Here, $h_{\mathrm{elem}}$ is the element size over the height with respect to the channel height $H_{0}$. The relative error at a time $t=10\lambda_{0}$ is defined as

(39) $$\epsilon \left(y\right)={\left.\frac{{\left(\int_{\Gamma_{\mathrm{out}}}{\left(\xi -\xi_{\mathrm{an}}\right)}^{2}\right)}^{1/2}}{{\left(\int_{\Gamma_{\mathrm{out}}}\xi_{\mathrm{an}}^{2}\right)}^{1/2}}\right|}_{t=10\lambda_{0}},$$

where ξ is the structure parameter on the outlet of the channel, $\xi_{\mathrm{an}}$ is the analytical solution, ϵ(y) is the relative error in ξ for different heights *y* on the outlet, and $\Gamma_{\epsilon }$ is $\Gamma_{\mathrm{out}}$.

**Figure 4:**
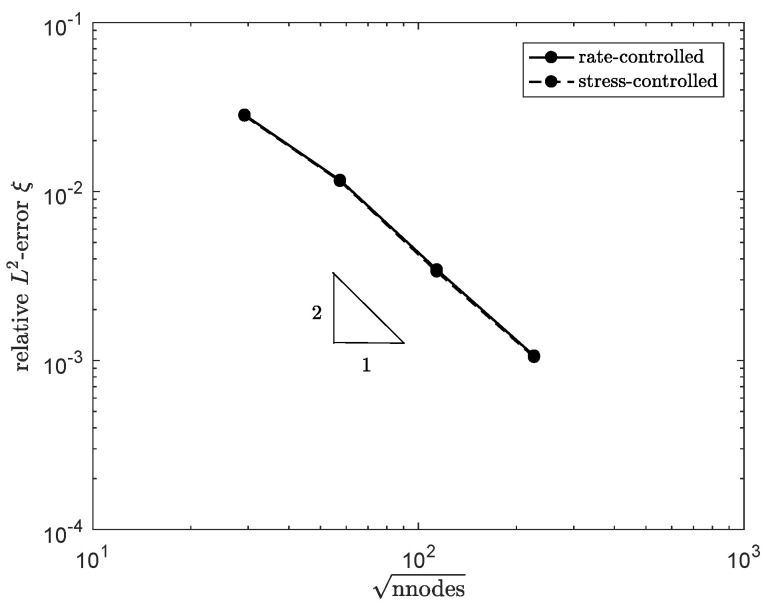
Relative error in ξ at the outlet of the channel for 2D meshes with different element sizes.

The result is shown in Figure 4. For both methods, convergence with order two is obtained, which was expected based on the order of interpolation of the elements.

**Figure 5:**
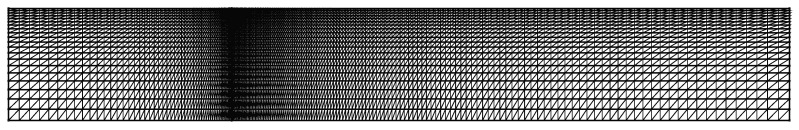
Two-dimensional planar swell mesh one uniform refinement step coarser compared to the mesh used throughout the remainder of this paper.

Figure 5 shows a 2D planar swell mesh that is one uniform refinement step coarser compared to the mesh used throughout the remainder of this paper. The coarsest element size at the symmetry axis of the mesh used in this paper is $h_{\mathrm{sym}}=0.05H_{0}$. The mesh is progressively refined with a factor of 5 towards the die wall $h_{\mathrm{wall}}=0.01H_{0}$, and with a factor of 10 towards the die exit $h_{\mathrm{die}-\mathrm{exit}}=0.005H_{0}$.

**Figure 6:**
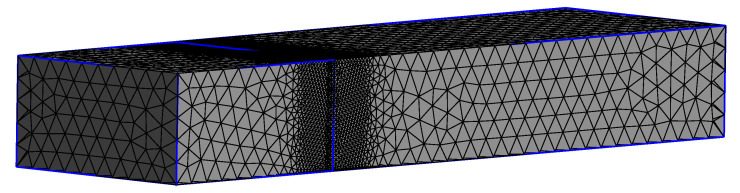
Three-dimensional mesh used in this study. Refinement factor at the die exit is 5.

The 3D problem is much more computationally demanding than the 2D planar problem. Therefore, a coarser mesh is used. The 3D mesh is shown in Figure 6. Here, the coarsest element size is $h_{\mathrm{sym}}=0.2H_{0}$. The mesh is refined with a factor 5 at the die exit. Since this mesh is much coarser than the 2D planar mesh, the 3D results will only be used to get a qualitative idea of the influence of thixotropy on the final extrudate shape. More information about the 2D and 3D swell mesh used in this paper can be found in Table 3.

#### 4.1.2. Time Convergence

**Figure 7:**
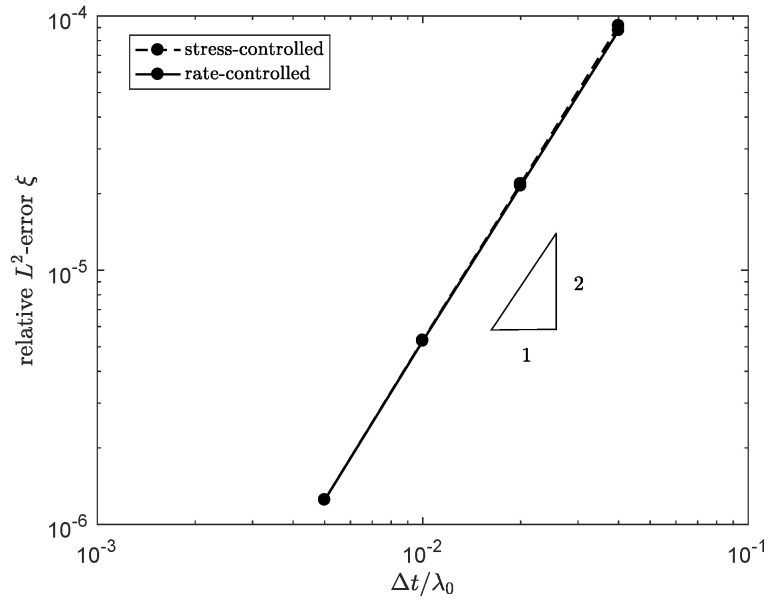
Relative error in ξ at the outlet of the channel at time $t=\lambda_{0}$, for different time step sizes Δt for the rate and stress-controlled approach.

Time convergence is tested on Mesh M4. The solution for different time step sizes is compared to a reference solution for a reference time step that is two times smaller compared to the smallest time step tested. The relative error at time $t=\lambda_{0}$ is calculated as follows:

(40) $$\epsilon \left(y\right)={\left.\frac{{\left(\int_{\Gamma_{\mathrm{out}}}{\left(\xi -\xi_{\mathrm{ref}}\right)}^{2}\right)}^{1/2}}{{\left(\int_{\Gamma_{\mathrm{out}}}\xi_{\mathrm{ref}}^{2}\right)}^{1/2}}\right|}_{t=\lambda_{0}},$$

where $\xi_{\mathrm{ref}}$ is the solution for the reference time step. The result is shown in Figure 7. For both methods, convergence with order two is obtained, with was expected based on the order of the time integration.

Throughout this paper, a time step size of $\Delta t=1.25\cdot 10^{-3}\lambda_{\mathrm{avg}}$ is used.

### 4.2. Constant Flow Rate

At first, a constant flow rate is applied to the inlet of the die. The influence of the model parameters in the rate and stress-controlled equations for the structure parameter ($\lambda_{\theta }$, $\gamma^{*}$, $\tau^{*}$) is studied in this section, as well as the difference between the rate and stress-controlled approach.

#### 4.2.1. Rheology

**Figure 8:**
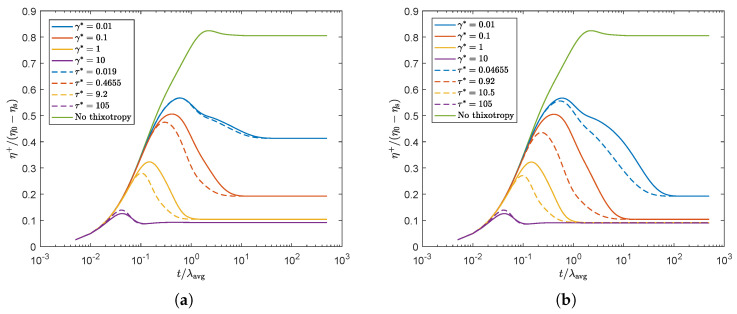
Transient polymer viscosity for ${\dot{\gamma }}_{c}$, where $\gamma^{*}$ and $\tau^{*}$ are chosen such that $\xi_{\mathrm{eq}}$ is matched for the rate and stress-controlled approach to obtain the same steady-state rheology for both models. (**a**) $\lambda_{\theta }=10\lambda_{\mathrm{avg}}$. (**b**) $\lambda_{\theta }=100\lambda_{\mathrm{avg}}$.

In order to study the differences between the rate and stress-controlled approach, the rheology of both methods has to be matched. To this end, we chose to match the equilibrium value for the structure parameter $\xi_{\mathrm{eq}}$ in steady shear (see Equation (38)) for both methods at a characteristic shear rate ${\dot{\gamma }}_{c}=U_{\mathrm{avg}}/H_{0}$. This characteristic shear rate is used in the simulations throughout this paper when the two models are compared and corresponds to a Weissenberg number of Wi=5. The transient polymer viscosity for two different recovery time scales $\lambda_{\theta }$ and different damage model parameters $\gamma^{*}$ and $\tau^{*}$ is shown in Figure 8. This figure shows that the steady state viscosity is indeed the same for the rate and stress-controlled approach for the characteristic shear rate, but the transient behaviors are not.

**Figure 9:**
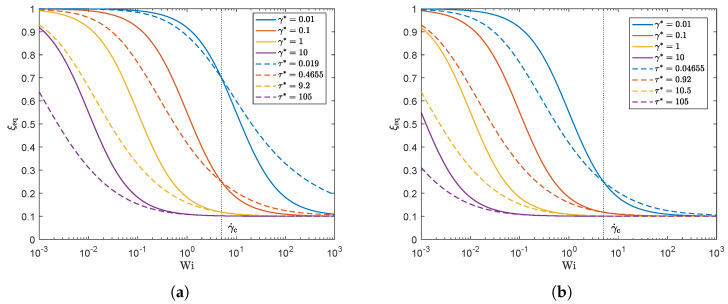
Equilibrium structure parameter $\xi_{\mathrm{eq}}$ as a function of Weissenberg number. Here, $\gamma^{*}$ and $\tau^{*}$ are chosen such that $\xi_{\mathrm{eq}}$ is matched for the rate and stress-controlled approach at a characteristic shear rate ${\dot{\gamma }}_{c}$. (**a**) $\lambda_{\theta }=10\lambda_{\mathrm{avg}}$. (**b**) $\lambda_{\theta }=100\lambda_{\mathrm{avg}}$.

The stress-controlled approach predicts a smaller overshoot compared to the rate-controlled approach for all damage parameters except the highest, for which a slightly higher overshoot is predicted by the stress-controlled approach. Figure 9 shows the equilibrium value of ξ in steady state for different Weissenberg numbers for both approaches. The vertical black dotted line indicates the Weissenberg number corresponding to the characteristic shear rate for which both models match. This figure shows that $\xi_{\mathrm{eq}}$ is indeed matched for the characteristic shear rate. It is however clear that for $\dot{\gamma }<{\dot{\gamma }}_{c}$, the stress-controlled approach predicts a smaller value for $\xi_{\mathrm{eq}}$ (indicating more structural damage), whereas for $\dot{\gamma }>{\dot{\gamma }}_{c}$, the stress-controlled approach predicts a larger value for $\xi_{\mathrm{eq}}$ (less structural damage) compared to the rate-controlled approach.

The damage parameters used in this paper to compare the stress and rate-controlled approach are given in Table 4.

#### 4.2.2. Influence Model and Model Parameters on Swell Behavior

In this section, we study the influence of different recovery time scales $\lambda_{\theta }$ and damage parameters $\gamma^{*}$, $\tau^{*}$, on the 2D planar swell behavior of thixotropic fluids. A constant flow rate *Q* is applied at the inlet such that Wi=5. The differences between the rate and stress-controlled approaches are presented. Results are compared for two Weissenberg numbers (Wi=1, Wi=5) for the rate-controlled approach with $\lambda_{\theta }=10\lambda_{\mathrm{avg}}$ and different damage parameters $\gamma^{*}$ to study the effect of elasticity.

**Figure 10:**
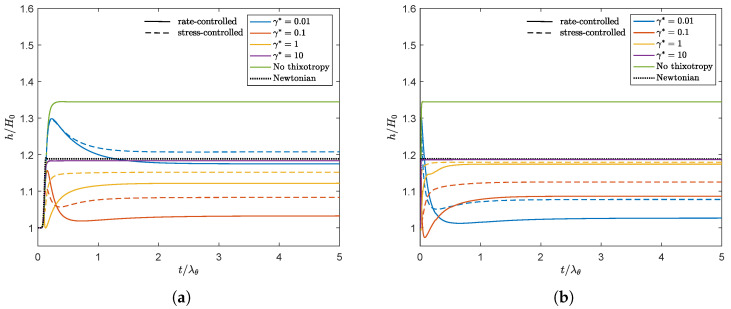
Swell ratio as a function of dimensionless time of the point of the free surface on $\Gamma_{\mathrm{out}}$ for different values of $\gamma^{*}$ for the rate-controlled approach and the corresponding value of $\tau^{*}$ for the stress-controlled approach. (**a**) $\lambda_{\theta }=10\lambda_{\mathrm{avg}}$ and (**b**) $\lambda_{\theta }=100\lambda_{\mathrm{avg}}$. Solid lines are the rate-controlled model predictions, dashed lines are the corresponding stress-controlled model predictions. The black dashed line indicates the Newtonian swell ratio. (**a**) $\lambda_{\theta }=10\lambda_{\mathrm{avg}}$. (**b**) $\lambda_{\theta }=100\lambda_{\mathrm{avg}}$.

Figure 10 shows the swell ratio of the point of the free surface on $\Gamma_{\mathrm{out}}$ in time for different model parameters and both the rate and stress-controlled models for Wi=5. The swell ratio for the viscoelastic fluid without thixotropy is also added, as well as the Newtonian swell ratio.

From this figure, the following trends can be observed:

- **Observation 1:** A larger damage parameter does not necessarily lead to a smaller swell ratio. Upon increasing the damage parameter, the results first show a swell ratio smaller than the swell ratio of a Newtonian fluid. Further increasing the damage parameter leads to a swell ratio approaching the value of a Newtonian fluid.
- **Observation 2:** The stress-controlled approach always results in a larger steady state swell ratio compared to the rate-controlled approach.
- **Observation 3:** For large values of the damage parameter ($\gamma^{*}$ and $\tau^{*}$), the swell ratio is higher when $\lambda_{\theta }$ is larger, whereas for small values of the damage parameter, the opposite effect is observed.
- **Observation 4:** For $\gamma^{*}=0.01$–0.1, the swell ratio of the outer point of the free surface shows a maximum. For $\gamma^{*}=1$, a maximum and a minimum in the swell ratio are observed.
- **Observation 5:** Thixotropy seems to always decrease the swell ratio compared to the case without thixotropy. This agrees with our expectations, since thixotropy decreases the elasticity in the material by decreasing the relaxation times. The swell ratio for the largest damage parameter tested approaches the swell ratio for a Newtonian fluid.

**Figure 11:**
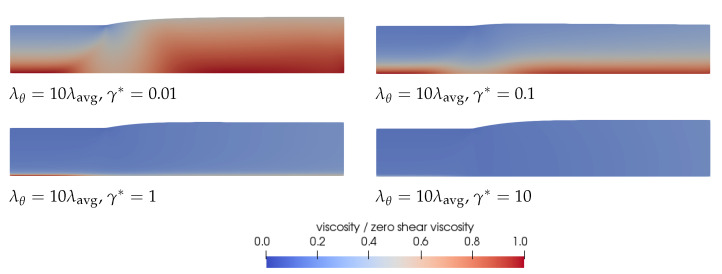
Total shear viscosity divided by the zero-shear viscosity predicted by the rate-controlled approach for $\lambda_{\theta }=10\lambda_{\mathrm{avg}}$ and different damage parameters $\gamma^{*}$.

**Figure 12:**
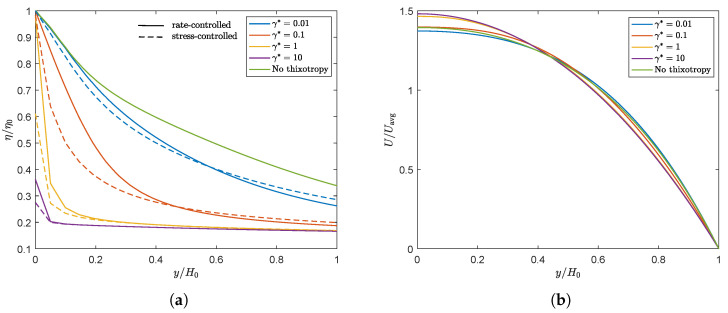
Ratio of the total viscosity and the zero-shear viscosity over the die height at $\Gamma_{\mathrm{in}}$ for $\lambda_{\theta }=10\lambda_{\mathrm{avg}}$ and different damage parameters for the rate and stress-controlled approaches (**a**). Dimensionless velocity magnitude over the die height at $\Gamma_{\mathrm{in}}$ for $\lambda_{\theta }=10\lambda_{\mathrm{avg}}$ and different damage parameters for the rate-controlled approach (**b**). (**a**) $\lambda_{\theta }=10\lambda_{\mathrm{avg}}$. (**b**) $\lambda_{\theta }=10\lambda_{\mathrm{avg}}$.

We will first focus on **Observation 1**. A smaller $\gamma^{*}$ leads to less damage and therefore to larger relaxation times and more elasticity in the material compared to a larger $\gamma^{*}$. Therefore, initially, a decrease in swell ratio is observed upon increasing the damage parameter. However, when we continue to increase $\gamma^{*}$, the steady state swell ratio starts to increase again. Since the relaxation times are adjusted with the local structure parameter ξ (See Equation (7)) but the moduli are kept constant, the viscosity $\eta_{p}=\sum_{k=1}^{m}G_{k}\left(\xi \lambda_{0,k}\right)$ also changes locally with ξ. The total shear viscosity $\eta =\eta_{s}+\eta_{p}$ divided by the zero-shear viscosity in the whole domain is shown in Figure 11 for $\lambda_{\theta }=10\lambda_{\mathrm{avg}}$ for the rate-controlled approach. This figure shows that upon increasing $\gamma^{*}$, first a low viscosity layer appears at the die wall. Upon further increasing the damage parameter, the viscosity difference in the die becomes smaller because the thickness of this low viscosity layer increases. The region with the highest viscosity is always in the middle of the die (at the symmetry line in Figure 11). The ratio of the total viscosity and the zero-shear viscosity is plotted over the height of the inlet of the die in Figure 12a. This figure shows that for small values of the damage parameter, there is a region of low viscosity fluid at to the die wall, but there is also a high viscosity region near the symmetry axis. Upon increasing the damage parameter, the thickness of this high viscosity core decreases until eventually there is only a small region of high viscosity fluid left near the symmetry axis of the die, approaching the result for a purely Newtonian fluid with a constant viscosity. The corresponding dimensionless velocity magnitude for the rate-controlled approach plotted over the die height at the inlet is shown in Figure 12b. This figure shows that the velocity profile is initially flattened with increasing $\gamma^{*}$, decreasing the swell ratio because the flow starts to look more like a plug flow. Upon further increasing $\gamma^{*}$, the thickness of the low viscosity layer increases, and the velocity profile becomes more parabolic again, leading to an increased swell ratio.

According to the inelastic swell theory presented by Tanner [19], a less viscous outer layer results in a decreasing swell ratio when the core region is large. This is also what is initially observed in Figure 10. However, **Observation 1** shows that when the high viscosity core becomes very small, the swell ratio starts to increase again and approaches the Newtonian swell ratio.

**Figure 13:**
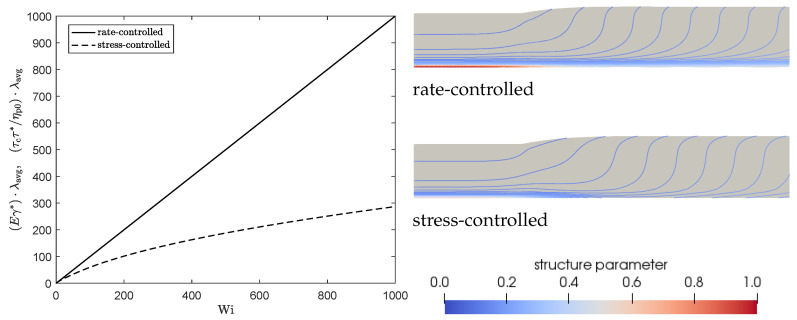
Damage terms of the rate and stress-controlled approaches as a function of Weissenberg number in steady shear (**left**). Contour plots of the structure parameter ξ for $\lambda_{\theta }=10\lambda_{\mathrm{avg}}$ an $\gamma^{*}=1$ for the rate-controlled approach and the corresponding values for the stress controlled approach (**right**). Unless indicated otherwise, all contour plots presented in this paper are made with equidistant contour lines with an interval of 0.01.

To explain **Observation 2**, we plotted the damage terms ($E\gamma^{*}$, $\tau_{c}\tau^{*}/\eta_{p0}$) as a function of the Weissenberg number in steady shear for both approaches in Figure 13 (left). This figure shows that, due to the dependency of $\tau_{c}$ on the local structure in the material, the damage term in the stress-controlled approach is not linearly dependent on the Weissenberg number, whereas this is the case for the rate-controlled approach. Figure 9 shows that this difference leads to a smaller structure parameter for small Weissenberg numbers but a higher structure parameter for high Weissenberg numbers for the stress-controlled approach. This is also shown in Figure 13 (right), where the contour lines for the structure parameter ξ are plotted for both approaches ($\lambda_{\theta }=10\lambda_{\mathrm{avg}}$, $\gamma^{*}=1$). Unless indicated otherwise, all contour plots presented in this paper are made with equidistant contour lines with an interval of 0.01. The stress-controlled approach predicts a smaller undamaged core compared to the rate-controlled approach. Figure 12 shows that for small values of the damage parameter, the viscosity close to the die wall is higher for the stress-controlled approach compared to the rate-controlled approach but smaller close to the symmetry axis. This leads to a flatter velocity profile for the rate-controlled approach and therefore to a larger swell ratio for the stress-controlled approach. For larger damage parameters, however, the high viscosity core even seems to be smaller for the stress-controlled approach compared to the rate-controlled approach. This leads to a larger maximum velocity and a more parabolic velocity profile in the die. This also causes the stress-controlled approach to predict a larger swell ratio.

**Figure 14:**
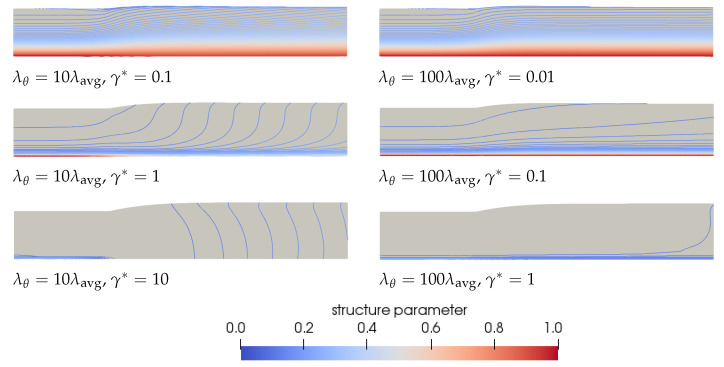
Contour plots of the structure parameter ξ predicted by the rate-controlled approach for $\lambda_{\theta }=10\lambda_{\mathrm{avg}}$ (**left**) and $\lambda_{\theta }=100\lambda_{\mathrm{avg}}$ (**right**) for different damage parameter and recovery time scale combinations that give the same $\xi_{\mathrm{eq}}$.

To explain **Observation 3**, we refer to Equation (38). This equation shows that the same equilibrium value of the structure parameter is found if the damage parameter times the recovery time scale is equal. This means that $\gamma^{*}=0.1$, $\lambda_{\theta }=10\lambda_{\mathrm{avg}}$ gives the same equilibrium structure as $\gamma^{*}=0.01$, $\lambda_{\theta }=100\lambda_{\mathrm{avg}}$. Figure 10 also shows that the swell ratio for the values that give the same $\xi_{\mathrm{eq}}$ are close but not equal. This can be explained by the different transient behavior for different recovery time scales $\lambda_{\theta }$. Figure 14 shows the contour plots for the structure parameter for $\lambda_{\theta }=10\lambda_{\mathrm{avg}}$ (left) and $\lambda_{\theta }=100\lambda_{\mathrm{avg}}$ (right) for different $\gamma^{*}\lambda_{\theta }$ combinations that give the same $\xi_{\mathrm{eq}}$.

From this figure, we can conclude that for large damage parameters, there is a large area in the die where the material has structure parameter $\xi =\xi_{\inf}$. This also shows that the layers of fluid with a certain structure parameter ξ extend over a greater length close to the free surface (contour lines are more horizontal) when $\lambda_{\theta }$ is larger. This can be attributed to the difference in transient behavior, because the time for the fluid to recover is longer. In the extrudate, the shear rates and the stresses are low, and therefore the structure can recover here. This will take longer when $\lambda_{\theta }$ is larger, meaning that the viscosity in the low viscosity layer away form the symmetry line will be lower for a longer time when $\lambda_{\theta }=100\lambda_{\mathrm{avg}}$. This leads to a larger velocity in the *x*-direction in the extrudate compared to $\lambda_{\theta }=10\lambda_{\mathrm{avg}}$ but a lower velocity in the *y*-direction. This leads to more extended layers of fluid with a certain structure parameter ξ.

**Figure 15:**
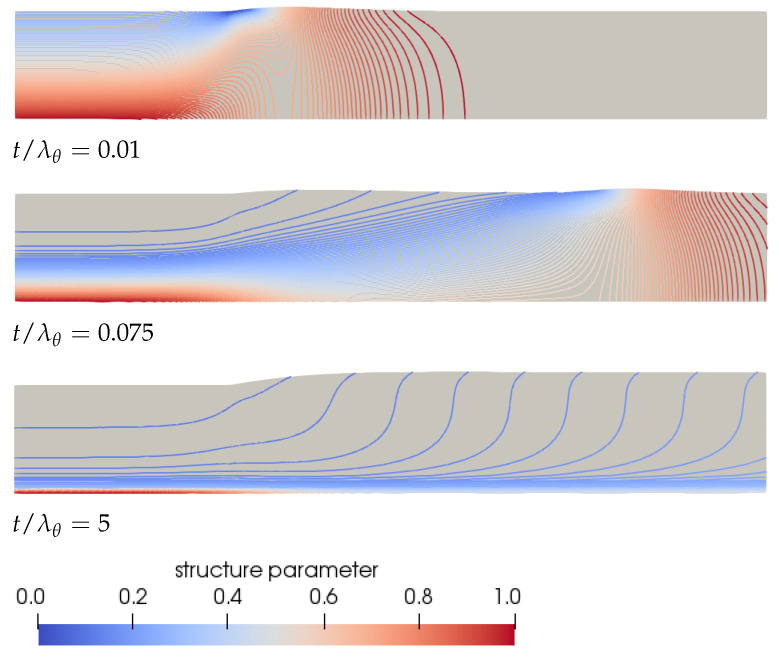
Contour plots of the structure parameter ξ predicted by the rate-controlled approach for $\lambda_{\theta }=10\lambda_{\mathrm{avg}}$ and $\gamma^{*}=1$ for different instances in time.

To explain **Observation 4**, we have to look at the transient behavior of the structure in the material. Initially, the fluid is undamaged (ξ=1). Due to flow, a small layer of damaged fluid starts to grow near the die wall. At this point, the fluid is still elastic and will start to swell once it leaves the die, leading to the maximum in the swell ratio. While the damaged layer of fluid starts to grow, a low viscosity and more viscous layer appears near the die wall, leading to a decrease in the swell ratio. After a certain amount of time, this layer spans almost the whole height of the die, leading to a small high viscosity core layer, and the swell ratio starts to increase again, leading to the minimum for $\gamma^{*}=1$. This is schematically shown in Figure 15 for $\lambda_{\theta }=10\lambda_{\mathrm{avg}}$, $\gamma^{*}=1$. This figure also shows the convection of the maximum and minimum swell height of the free surface.

**Figure 16:**
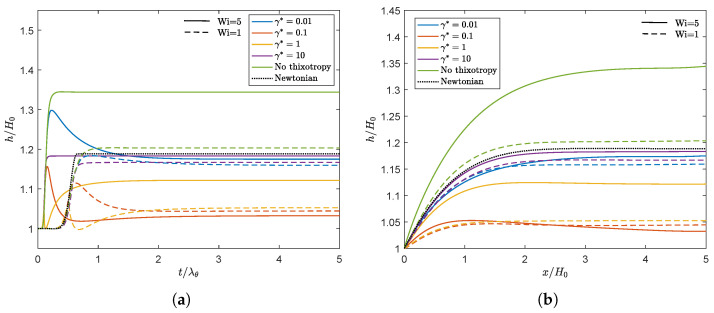
Swell ratio as a function of dimensionless time for the point of the free surface on $\Gamma_{\mathrm{out}}$ (**a**) and final swell ratio of the free surface as a function of the *x*-coordinate along the free surface (**b**). Here, $x/H_{0}=0$ corresponds to the *x*-coordinate at the die exit. Results are obtained for different values of $\gamma^{*}$ for the rate-controlled approach using two different Weissenberg numbers. Solid lines indicate the results for Wi=5, whereas dashed lines indicate the results for Wi=1.

To show the influence of the Weissenberg number, simulations are also performed for Wi=1. Figure 16a shows the swell ratio in time for $\lambda_{\theta }=10\lambda_{\mathrm{avg}}$ and different damage parameters for the rate-controlled equation. The result shows the same qualitative trend as Figure 10 for increasing $\gamma^{*}$. The overall swell ratio is higher for a higher Weissenberg number due to the larger effect of elasticity in the material. For both values of Wi, it is observed that upon increasing the damage parameter, first, a swell ratio smaller than the Newtonian value is obtained. Upon further increasing $\gamma^{*}$, the swell ratio approaches the Newtonian value. $\gamma^{*}=0.1$ is the only damage parameter for which a lower final swell ratio is predicted for Wi=5 compared to Wi=1. Figure 16b shows that the final swell ratio for this damage parameter close to the die exit ($x/H_{0}=0$) is higher for Wi=5 compared to Wi=1, whereas near $\Gamma_{\mathrm{out}}$ ($x/H_{0}=5$), the opposite is observed. Therefore, we suspect that there is a complex interplay between higher swell due to a higher Weissenberg number and a decrease in swell due to larger damage at higher Wi for $\gamma^{*}=0.1$ in the extrudate. More research is needed to fully understand this phenomenon.

#### 4.2.3. Three-Dimensional Extrudate Swell

**Figure 17:**
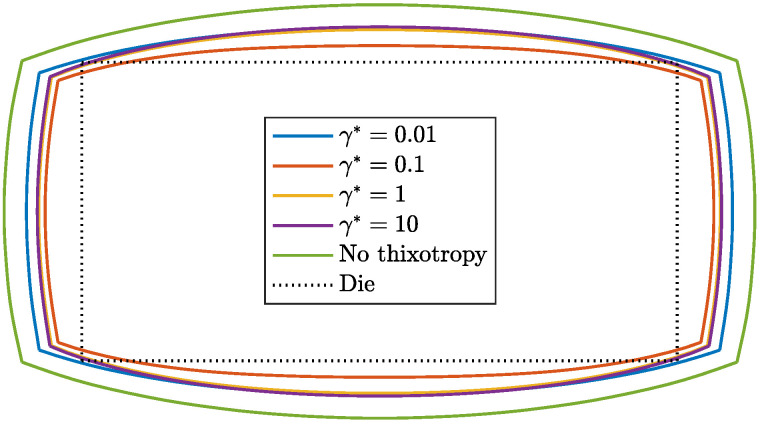
Contour of a 3D extrudate of a thixotropic viscoelastic fluid. Evolution of the structure in the material is modeled using the rate-controlled approach with $\lambda_{\theta }=10\lambda_{\mathrm{avg}}$ and different damage parameters for Wi=5.

Three-dimensional simulations of a viscoelastic fluid emerging from a rectangular die with an aspect ratio of 2:1 are performed to show the effect of a changing structure in the material in three-dimensional extrudates. The rate-controlled approach is used to model the time-dependent evolution of the structure in the material. Figure 17 shows the contour of the extrudate shape in steady state for $\lambda_{\theta }=10\lambda_{\mathrm{avg}}$, different damage parameters and Wi=5. Here, a similar effect of thixotropy on extrudate swell as for the 2D planar problem is observed.

Initially, the swell decreases when thixotropy is introduced. For increasing $\gamma^{*}$, however, the swell ratio starts to increase again. This effect is more pronounced for the height of the extrudate than the width of the extrudate. This can be attributed to the higher shear rate in the height direction compared to the width direction. The swell ratios for a thixotropic fluid are always smaller compared to the viscoelastic fluid without thixotropy.

### 4.3. Fluctuating Flow Rate

All previous results are obtained by applying a constant flow rate *Q* at the die inlet. However, in many industrial processes, the flow rate is not constant but fluctuates in time. In this section, the effect of a fluctuating flow rate on the structure in the material and the swell ratio of the 2D planar extrudate is studied using the rate-controlled model for the kinetic equation of the structure parameter ξ. A sinusoidal flow rate is applied to the inlet, with different periods $2\pi /t_{p}$, where $t_{p}$ is the time it takes for the sine function to complete one period:

(41) $$Q=\sin\left(2\pi \frac{t}{t_{p}}\right)+U_{\mathrm{avg}}H_{0}.$$

**Figure 18:**
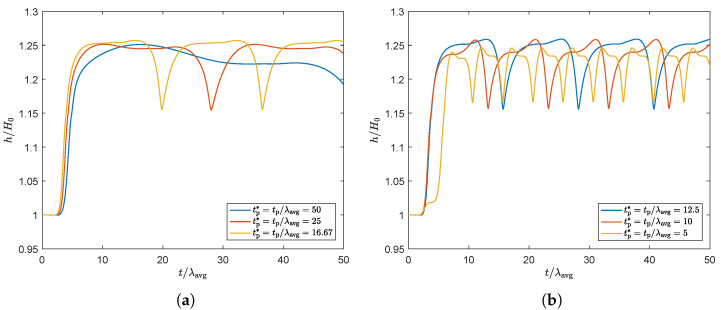
Swell ratio as a function of dimensionless time for the point of the free surface on $\Gamma_{\mathrm{out}}$, for a sinusoidal flow rate with dimensionless frequency $f^{*}=1/t_{p}^{*}$, with $t_{p}^{*}=t_{p}/\lambda_{\mathrm{avg}}$.

First, the effect of a fluctuating flow rate is studied for the non-thixotropic fluid and an average Weissenberg number of Wi=1. Here, flow rates are applied with different dimensionless times for the period of a sine $t_{p}^{*}=t_{p}/\lambda_{\mathrm{avg}}$. The swell ratio of the outer point on the free surface at $\Gamma_{\mathrm{out}}$ is shown as a function of dimensionless time in Figure 18. This figure shows that, although the flow rate is sinusoidal, the corresponding periodicity in the swell ratio is presented by a higher-order sine function, where two frequencies are visible. An explanation for this is as follows: for high flow rates, more fluid is flowing through the die at a certain time interval compared to lower flow rates. Therefore, it takes longer for the effect of the decrease in flow rate to be noticed at the end of the extrudate compared to the effect of the increase in flow rate, and the effect of two different time scales is observed.

**Figure 19:**
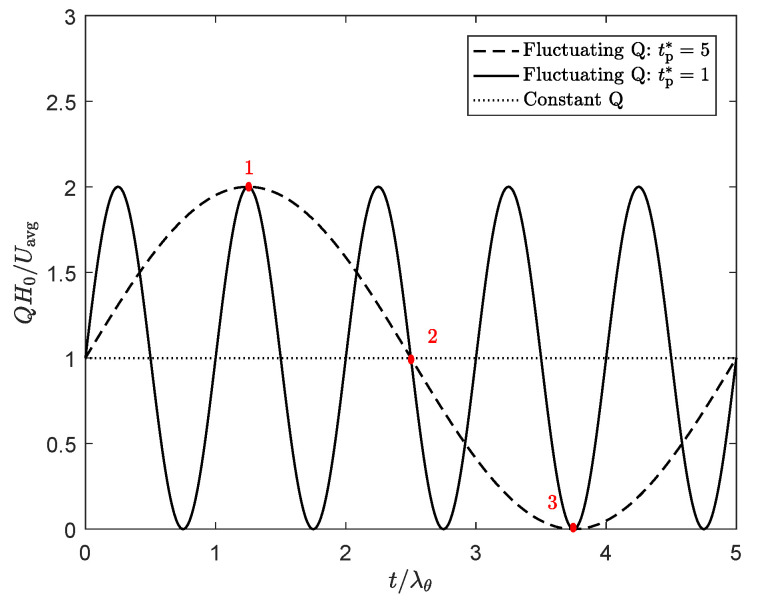
Sinusoidal flow rate with dimensionless period $t_{p}^{*}=t_{p}/\lambda_{\theta }=1$ (solid line), and $t_{p}^{*}=t_{p}/\lambda_{\theta }=5$ (dashed line) and the constant flow rate applied in previous results (dotted line).

The frequencies chosen to study the influence of thixotropy on extrudate swell for a fluctuating flow rate correspond to the following dimensionless times for one period of the sine: $t_{p}^{*}=t_{p}/\lambda_{\theta }=1$, $t_{p}^{*}=t_{p}/\lambda_{\theta }=5$, as shown in Figure 19. Results are obtained for $\lambda_{\theta }=10\lambda_{\mathrm{avg}}$, different damage parameters and an average Weissenberg number of Wi=1.

**Figure 20:**
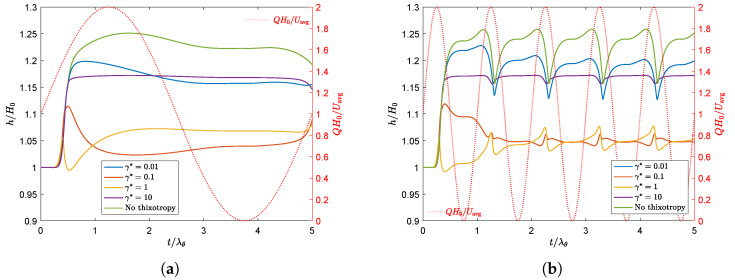
Swell ratio as a function of dimensionless time for the point of the free surface on $\Gamma_{\mathrm{out}}$, for different values of $\gamma^{*}$ and a sinusoidal flow rate with dimensionless frequency $f^{*}=1/t_{p}^{*}$, with $t_{p}^{*}=t_{p}/\lambda_{\theta }=5$ (**a**), and $t_{p}^{*}=t_{p}/\lambda_{\theta }=1$ (**b**). The red dotted line represents the applied flow rate.

Figure 20 shows the swell ratio of the point of the free surface on $\Gamma_{\mathrm{out}}$ as a function of dimensionless time for a fluctuating flow rate with both dimensionless periods $t_{p}^{*}$. The swell ratio is plotted for different values of $\gamma^{*}$.

**Figure 21:**
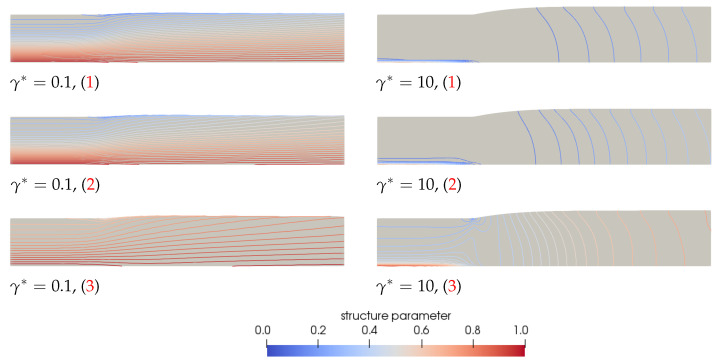
Contour plots of the structure parameter ξ predicted by the rate-controlled approach for $\lambda_{\theta }=10\lambda_{\mathrm{avg}}$ and $\gamma^{*}=0.1$ (**left**) and $\gamma^{*}=10$ (**right**) for different instances in time as indicated by the red numbers in Figure 19 for a fluctuating flowrate Q. The contour plots in this figure are made with equidistant contour lines with an interval of 0.025 for clarity.

This figure shows that for a fluctuating flow rate, the fluctuation is also visible in the swell ratio of the end point of the free surface. For small values of $\gamma^{*}$ and non-thixotropic materials, the swell ratio increases with *Q*, due to an increase in the Weissenberg number, and decreases when the flow rate decreases. Since the material starts to swell more when the flow rate increases, and it takes time for this material to reach the end of the extrudate, there is a delay between the maximum in the flow rate and the corresponding maximum in the swell ratio for small values of $\gamma^{*}$ and the non-thixotropic fluid. For the high frequency case, the fluctuations in the swell ratio are much more pronounced compared to the small frequency case. The fluctuations seem to weaken when the damage parameter increases. It can also be observed that, for the high frequency case, the maxima in the swell ratio for $\gamma^{*}=1$ and $\gamma^{*}=10$ occur at the same dimensionless time as the minima for $\gamma^{*}=0.01$ and the non-thixotropic fluid. For higher values of $\gamma^{*}$, the structure parameter will be smaller when *Q* is large due to an increasing shear rate, meaning that the structure in the material is more damaged and the fluid becomes less elastic. This decreases the swell ratio. When *Q* decreases again, the structure in the material increases, hence increasing the elasticity and thus the swell ratio. The complex coupling of the competing effect of decreasing Wi due to damaging the structure at high flow rates, while simultaneously increasing the shear rate at higher flow rates and therefore increasing Wi, leads to the complex transient behavior for a fluctuating flow rate. To show the change in structure in the material, the contour of the structure parameter ξ is shown in Figure 21 for $\gamma^{*}=0.1$ and $\gamma^{*}=10$ at a time interval where the flow rate is at its maximum (1), the flow rate equals the constant applied flow rate (2) and where the flow rate is at its minimum (3), as indicated in Figure 19. Figure 21 clearly shows the change in structure at the different moments in time, due to the fluctuating flow rate. When the flow rate is at its maximum (1), there is a large area of damaged material (small structure parameter ξ) in areas where the shear rate is high. This is much more severe when the damage parameter is large. When the flow rate decreases again, the material has time to recover, and the structure parameter will increase again.

## 5. Conclusions

In this paper, we studied the effect of thixotropy on extrudate swell. To this end, we used a rate and stress-controlled equation for a structure parameter that indicates the degree of structure in the material. The relaxation times are adjusted with this structure parameter, which effectively means that the fluid becomes less elastic when the structure is damaged.

Rate and stress-controlled approaches are compared by matching the damage parameters in the evolution equation for the structure parameter in such a way that the steady state structure parameter and the steady state viscosity are matched at a characteristic shear rate. Furthermore, the effects of the recovery time scale and the damage parameter in the kinetic equation for the structure parameter are studied. It was found that thixotropy in general decreases the swell ratio. When the damage parameter in the models is increased, an outer layer with lower viscosity appears near the die wall, which decreases the swell ratio to a value below the Newtonian swell ratio. Upon further increasing the damage parameter, the high viscosity core becomes very small, leading to an increase in the swell ratio approaching the Newtonian value. Furthermore, it was found that the stress-controlled approach always predicts a larger swell ratio compared to the rate-controlled approach for the Weissenberg number range studied in this paper.

Results for varying the recovery time scale of the structure in the material show that even though the damage and recovery parameters predict the same equilibrium value for ξ, a different transient behavior of the structure is observed. This leads to different, but comparable, swell ratios for the same equilibrium structure parameter.

The effect of thixotropy on the swell ratio is studied for two different Weissenberg numbers (Wi=1, Wi=5). Results show an overall higher swell ratio for the higher Weissenberg number. However, a complex interplay between higher swell due to a higher Weissenberg number and a decrease in swell due to larger damage at higher Wi is observed for small damage parameters. This interplay is an interesting topic for future research.

A proof of concept of the thixotropy model is given in 3D by performing simulations for Wi=5 using the rate-controlled approach and different damage parameters. Contours of the final extrudate shape show a similar effect of thixotropy on extrudate swell compared to the 2D planar problem.

Finally, the effect of a fluctuating flow rate is studied for the rate-controlled approach. To this end, a sinusoidal flow rate is applied with different frequencies. Results show that the fluctuation in the swell ratio decreases with increasing damage parameter. There also seems to be a complex coupling between the decrease of Wi when the structure is damaged at high flow rates and the increase in Wi due to an increasing shear rate at increasing flow rates. This leads to complex transient behavior.

In conclusion, this paper shows that thixotropy in general decreases extrudate swell, but complex transient behavior occurs when the fluid is thixotropic. Results show that the emergence of fluid layers with different viscosities in the die due to thixotropy highly influences the swell ratio.

## Figures and Tables

**Table 1:** Material parameters used in this study. Additionally, the parameters β=0.1 and $\xi_{\inf}=0.1$ are used throughout this paper.

Mode	$\lambda_{0,k}/\lambda_{\mathrm{avg}}$	$G_{0,k}\lambda_{\mathrm{avg}}/\eta_{p0}$	α
1	10	0.0048	0.3
2	1	0.48	0.3
3	0.1	4.8	0.3

**Table 2:** Meshes used in the mesh convergence study on a 2D channel flow problem.

Mesh	# Nodes	$h_{\mathrm{elem}}/H_{0}$
M1	7209	0.25
M2	27217	0.125
M3	105633	0.0625
M4	416065	0.03125

**Table 3:** Two and three-dimensional meshes for the swell problems in this paper.

Mesh	# Nodes	# Elements	$h_{\mathrm{sym}}/H_{0}$	$h_{\mathrm{wall}}/H_{0}$	$h_{\mathrm{die-exit}}/H_{0}$
2D	58401	28800	0.05	0.01	0.005
3D	50467	32444	0.2	0.2	0.04

**Table 4:** Damage parameters used to match the rheology of the rate-controlled approach ($\gamma^{*}$) and the stress-controlled approach ($\tau^{*}$) at the characteristic shear rate ${\dot{\gamma }}_{c}$ for two different recovery time scales.

$\lambda_{\theta }=10\lambda_{\mathrm{avg}}$		$\lambda_{\theta }=100\lambda_{\mathrm{avg}}$	
$\gamma^{*}$	$\tau^{*}$	$\gamma^{*}$	$\tau^{*}$
0.01	0.019	0.01	0.04655
0.1	0.4655	0.1	0.92
1	9.2	1	10.5
10	105	10	105
